# One-dimensional computational circulatory models: a scoping review

**DOI:** 10.31744/einstein_journal/2026RW1854

**Published:** 2026-03-30

**Authors:** Gabriella de Araujo Cunha Lima Nóbrega, Stefano Garzon, Pablo J. Blanco, Pedro Alves Lemos

**Affiliations:** 1 Hospital Israelita Albert Einstein São Paulo SP Brazil Hospital Israelita Albert Einstein, São Paulo, SP, Brazil.; 2 Università degli Studi di Roma Rome Italy Università degli Studi di Roma (La Sapienza), Rome, Italy.; 3 Laboratório Nacional de Computação Científica Petrópolis RJ Brazil Laboratório Nacional de Computação Científica, Petrópolis, RJ, Brazil.

**Keywords:** Mathematical computing, Hemodynamics, Cardiovascular system, Models

## Abstract

**Background:**

Computational modeling of human circulatory system has evolved significantly in recent decades. Among the various modeling strategies, one-dimensional (1D) models have emerged as alternatives to more complex models because of their balance between physiological accuracy and computational efficiency.

**Objective:**

This scoping review aimed to summarize and compare the studies on 1D computational models of the entire circulatory system, including those that incorporated additional 0D and 3D components.

**Methods:**

A systematic search was performed for studies on computational 1D models of the entire arterial tree. Studies were eligible if they employed 1D modeling either exclusively or in combination with 0D and/or 3D components. Article screening, data extraction, and analyses were conducted in accordance with the PRISMA-ScR guidelines.

**Results:**

Out of the 6,841 records, 19 studies were included. Eleven articles presented strictly 1D nonlinear models, two used linear 1D models, and six employed multiscale frameworks that integrated 1D, 0D, and/or 3D components. Nonlinear 1D models consistently outperformed linear models in simulating large elastic arteries and pathological conditions, whereas linear models were effective in simulating small vessels under low-pressure variations. Multiscale models improve local hemodynamic details, but impose significantly higher computational costs.

**Conclusion:**

1D models provide a robust and computationally efficient framework for simulating global cardiovascular hemodynamics. Although nonlinear and multiscale models enhance the physiological fidelity and adaptability to complex scenarios, their higher computational demands should be weighed against the available resources and specific clinical or research goals.

## INTRODUCTION

The circulatory system is highly complex, both anatomically and functionally, and it is among the most challenging systems in the human body to comprehend. The dynamic interaction between blood vessels and the heart, along with regional circulation and constant metabolic variations, renders studying the circulatory system challenging.^( 1 )^ Recently, significant advances in imaging and scientific computing have provided more detailed analyses of the physiological functions of the circulatory system.^( 2 )^

Growing computational capacity enables the integration of actual hemodynamic parameters with mathematical models of the circulatory system.^( 3 , 4 )^ Conversely, the intrinsic multifactorial nature of the human cardiovascular system may render simulation models limitless and complex. In one-dimensional (1D) modeling of the circulatory system, linear models assume a proportional relationship between pressure and flow, simplifying the governing equations and making them computationally efficient. However, this linearity limits their ability to simulate complex behaviors such as wave reflections or pressure-dependent vessel compliance. In contrast, nonlinear models incorporate these phenomena by considering the viscoelastic properties of the vessel walls and the complex interactions between flow and pressure, providing more realistic simulations of hemodynamics, particularly under pathological conditions. In addition, zero-dimensional (0D) models or lumped-parameter models represent the cardiovascular system as distinct compartments connected by resistance, compliance, and inertance. These models are useful for simulating global circulatory behavior or for coupling with 1D and 3D models, although they lack spatial resolution. Therefore, producing accurate 1D models of the circulatory system to provide adequate simulations at lower computational costs has been focused on.^( 5 , 6 )^

However, retrieving the different models from literature and comparing them is also challenging.

## OBJECTIVE

The objective of this scoping review was to condense and compare various 1D computational models of the entire circulatory system.

## METHODS

### Study design and search strategies

This scoping review was conducted in accordance with the Preferred Reporting Items for Systematic reviews and Meta-Analyses (PRISMA) extension for Scoping Reviews (PRISMA-ScR).^( 7 )^We searched electronic databases (PubMed and Embase) for articles published in English that used 1D computational models of the entire circulatory tree. The search strategy is detailed in Table 1S , 2S and 3S, Supplementary Material. The reference lists of the retrieved articles were also screened for eligible studies. All references were exported to and reviewed using EndNote (version 20.6; Clarivate, PA, USA). After omitting duplicates, we proceeded with the screening process in a stepwise manner as follows: 1) In the first screening phase, we excluded articles based on title review - the absence of relevance to computational modeling or circulatory system structure was the main criteria; 2) In the second phase, we read the abstracts of the remaining articles, and articles were excluded for not meeting the inclusion criteria, such as lack of whole system modeling or absence of 1D elements; and 3) the remaining studies were selected for full-text analysis and inclusion. Studies were listed based on thematic grouping and relevance to the classification structure (linear, nonlinear, and multiscale) rather than chronological order.

**Table 1S:** Search strategies in different databases

Database	Search string	Articles retrieved
PubMed	(“Hemodynamics”[MeSH Terms] OR “hemodynamics”[tw] OR “blood flow”[tw]) AND (“Cardiovascular System”[MeSH Terms] OR “arterial network”[tw] OR “vascular tree”[tw] OR “arterial system”[tw]) AND (“Models, Cardiovascular”[MeSH Terms] OR “data assimilation”[tw] OR “computational model”[tw] OR “mathematical model”[tw] OR “1D model”[tw] OR “one-dimensional model”[tw] OR “reduced-order model”[tw] OR “lumped parameter model”[tw]) AND (“Simulation”[MeSH Terms] OR “simulation”[tw])	3,757
Embase	(‘hemodynamics’/exp OR ‘hemodynamics’:ti,ab,kw,de,dn,df,mn,tn OR ‘blood flow’:ti,ab,kw,de,dn,df,mn,tn) AND (‘cardiovascular system’/exp OR ‘arterial network’:ti,ab,kw,de,dn,df,mn,tn OR ‘vascular tree’:ti,ab,kw,de,dn,df,mn,tn OR ‘arterial system’:ti,ab,kw,de,dn,df,mn,tn) AND (‘biological model’/exp OR ‘data assimilation’:ti,ab,kw,de,dn,df,mn,tn OR ‘computational model’:ti,ab,kw,de,dn,df,mn,tn OR ‘mathematical model’:ti,ab,kw,de,dn,df,mn,tn OR ‘1d model’:ti,ab,kw,de,dn,df,mn,tn OR ‘one-dimensional model’:ti,ab,kw,de,dn,df,mn,tn OR ‘reduced-order model’:ti,ab,kw,de,dn,df,mn,tn OR ‘lumped parameter model’:ti,ab,kw,de,dn,df,mn,tn) AND (‘simulation’/exp OR ‘simulation’:ti,ab,kw,de,dn,df,mn,tn)	3,061

### Eligibility

Studies were considered eligible for inclusion if they used computational simulations of the entire arterial circulatory tree using 1D computational models. Studies were also eligible if they used other dimensional models (such as 0D or 3D) in addition to a 1D model.

### Data extraction

The selected articles were retrieved in full and their findings were summarized in a standard form containing the study objective, model characteristics (including model type, wall properties, geometry, and validation methods), number of arterial segments, inclusion of the venous system in the model, key results, and conclusions. The standard form and individual study summaries are provided in the Supplementary Material.

## RESULTS

**Figure 1:**
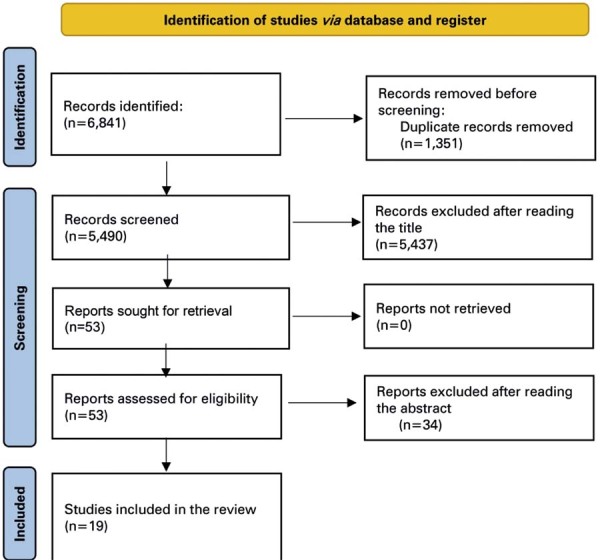
Study selection flowchart

The search strategy yielded 6,184 articles. Of these, 1,351 were duplicates and were excluded. The titles of the remaining 5,490 articles were read and 5,437 were excluded. The abstracts of the remaining 53 articles were fully read, and 19 articles that met the scope of our review were finally selected, retrieved, and read in full. A complete flowchart of the search strategy is shown in figure 1 . We summarized the findings of the selected articles based on model type (linear 1D, nonlinear 1D, and multiscale). We also provided a summary of the findings of these articles - according to author, year, model type, number of arterial segments, inclusion of the venous system, and main features of each model - in table 1 .

**Table 1:** One-dimensional computational models of the cardiovascular system

Author (year)	Model type (1D/Others)	Number of arterial segments	Includes venous system	Main features
Alastruey et al. (2011)^(^^10^^)^	1D nonlinear	37	No	*In vitro* validation, comparison with elastic model, pulse wave propagation analysis
Avolio (1980)^(^^11^^)^	1D	128	No	Multi-branched model with realistic arterial properties, impedance and wave reflection analysis
Bárdossy et al. (2010)^(^^12^^)^	1D	45	No	Method of characteristics (MOC), arteriosclerosis simulation
Blanco et al. (2013)^(^^18^^)^	1D + 0D + 3D	128	Yes (lumped 0D)	Closed-loop model; cardiac valves, stenosis and regurgitation modeling; local 3D coupling
Blanco et al. (2014)^(^^13^^)^	1D	2,142	No	Anatomical and physiological criteria for flow distribution, terminal model calibration
Blanco et al. (2015)^(^^6^^)^	1D	2,142	No	High anatomical resolution, parameter sensitivity, generic and patient-specific simulation
Blanco et al. (2020)^(^^14^^)^	1D	86 (simplified model) 2,142 (detailed model)	No	Comparison between simplified and detailed networks, anatomical impact on simulations
Liang et al. (2009)^(^^19^^)^	1D + 0D	55	Yes (lumped 0D)	Closed-loop multiscale model; study of aortic and arterial stenoses in different locations
Müller et al. (2014)^(^^20^^)^	1D + 0D	85	Yes (detailed 1D)	Emphasis on venous collapsibility, anatomical variability; validation with MRI data
Müller et al. (2023)^(^^15^^)^	1D	2,185	Yes (detailed 1D)	Closed model with high anatomical detail; focus on cerebral and coronary circulation
Mynard et al. (2008)^(^^16^^)^	1D	approximately 40	No	Integration of ventricle, aortic valve, and coronary flow using Galerkin method
Mynard et al. (2015)^(^^21^^)^	1D + 0D	396 (arterial + venous)	Yes	Closed and complete adult cardiovascular model, including cardiac interactions
Olufsen (1999)^(^^17^^)^	1D	21 large arteries + structured tree outflow (approximately 17 generations each)	No	Structured outflow boundary conditions; validation with real data
Reymond et al. (2009)^(^^22^^)^	1D	103	No	Includes cerebral circulation; validation with *in vivo* data
Safaei et al. (2016)^(^^32^^)^	1D + 0D + 3D	86	Yes	Collaborative platform with 1D/3D integration, OpenCMISS; customizable model
Schaaf et al. (1972)^(^^33^^)^	1D	47	No	Simulation with nonlinear characteristics and method of characteristics
Stergiopulos et al. (1992)^(^^34^^)^	1D	55	No	Complete model with arterial and aortic stenoses
Wang et al. (2004)^(^^8^^)^	1D	55	No	Method of characteristics; focus on wave reflection in complex geometries
Westerhof et al. (2020)^(^^9^^)^	1D	121	No	Model for ages 0-20 years; assessed flow and pressure relations across age groups, including anatomical development

### Linear 1D models

Wang et al **.** explored the role of wave reflections and re-reflections in the systemic arterial system using a linearized 1D model of 55 large arteries - isolating the effect of arterial geometry on wave dynamics while simplifying the cardiac input and eliminating nonlinearities - to better understand the pressure and velocity waveforms in normal and pathological scenarios. The model was validated against *in vivo* and literature data. Their main findings include: (i) re-reflections at bifurcations are the main contributors to waveforms, (ii) their algorithm tracks waves precisely, and (iii) distal waveform changes and pathological alterations ( *e.g.* , occlusion and regurgitation) can be explained by wave behavior alone.^( 8 )^

Westerhof et al. developed a distributed hemodynamic model of the human arterial tree that accounts for the developmental changes from newborns to adults. The model comprised 121 arterial segments, and compared simulated and *in vivo* measurements. The authors demonstrated that the peripheral pressure in children aged <5 years is approximately the central pressure and that the amplification and pulse wave velocity increased with age.^( 9 )^

### Nonlinear 1D models

Alastruey et al. built a model to assess the accuracy of nonlinear 1D viscoelastic equations of pressure and flow wave propagation. The authors also explored a metric called the harmonic flow error, which refers to the difference between the simulated and measured harmonic components (i.e., frequency content) of blood flow waveforms. It was calculated by decomposing the flow signal into its frequency components using Fourier analysis and comparing the amplitude and phase of each harmonic. This error metric allows a detailed assessment of how accurately a model reproduces the shape and dynamics of pulsatile blood flow beyond simple mean flow or peak values. The model was based on 1D nonlinear time-domain viscoelastic equations and compared with *in vitro* measurements from a 1:1 replica of 37 conduit arteries with a simulated fluid mimicking blood. When compared with the purely elastic models, the model performed significantly better, with lower pressure (2.5% *versus* 3.0%, p<0.01), flow rate (10.8% *versus* 15.7%, p<0.01), and harmonic flow errors (3.3% *versus* 7.0%, p<0.01). They concluded that i) including wall viscoelasticity significantly improved the accuracy of 1D simulations, and ii) 1D viscoelastic modeling achieved a good balance between accuracy and computational cost.^( 10 )^

Avolio presented a realistic, multi-branched 1D computational model of the entire human arterial system. This model accounted for wave propagation, impedance, and pathological states, such as arteriosclerosis and arterial stenosis. Using an arterial network of 128 segments, this model yielded accurate simulations of the pressure and flow dynamics in the circulatory tree, in agreement with the experimental data.^( 11 )^

Bárdossy et al developed a 1D arterial model that incorporated the Stuart viscoelastic model to describe the mechanical behavior of arterial walls. This model accounted for three key components: instantaneous elasticity, capturing the immediate response of the wall to pressure; viscous damping, modeling energy dissipation and wave attenuation; and history-dependent deformation, which reflects the time-evolving strain response because of the viscoelastic nature of the vessel wall. This time-dependent component allowed the model to reproduce realistic arterial behavior under pulsatile flow, including wall hysteresis and the dynamic pressure-diameter relationship. The arterial network comprised 45 viscoelastic segments and successfully replicated physiological waveforms, such as backflow in the iliac arteries during diastole and the dicrotic notch in the aortic pressure curves. The model was also capable of simulating localized stenoses and their hemodynamic effects.^( 12 )^

Blanco et al. devised this study to accurately define criteria for blood flow distribution in preparation for their detailed 1D arterial model (ADAN - anatomically detailed arterial network). They developed a numerical calibration algorithm to compute the terminal resistance, allowing their model of 2,142 arteries (1,598 arterial segments and 544 perforating arteries) to simulate the regional perfusion across 144 vascular beds (specific organs and territories). Their model provided a high-resolution arterial network with validated blood flow distribution criteria, accounted for specific and distributed perfusion using advanced anatomical data, and was suitable for regional hemodynamic studies and surgical planning.^( 13 )^

Blanco et al. presented a detailed 1D computational model, ADAN, which was calibrated in their previous work.^( 13 )^ This is a model of the entire arterial system of an average adult male that integrates vascular anatomy, morphometry, wall mechanics, and hemodynamics. The model was validated against both generic physiological data and patient-specific measurements. The model featured 2,142 arteries and blood supply to 28 specific organs and 116 vascular territories, with arterial wall properties based on a viscoelastic model that includes collagen distribution in the walls. However, this model did not include the venous system. Pressure and flow waveforms matched *in vivo* measurements across the central and peripheral arteries, and the model produced cardiovascular indices, such as heart rate and cardiac output, all within normal ranges.^( 6 )^

Blanco et al. further developed a simplified version of the ADAN model with 86 arterial segments (ADAN86), and compared its predictive capacity with that of the full model with >2,000 arteries. The model properties remained the same, and the reduced model was compared with the complete ADAN model and those in the literature. Although the ADAN86 model performed adequately under healthy conditions, it outperformed the full ADAN model in the simulations of pathological conditions.^( 14 )^

Müller et al. presented a model derived from the ADAN framework, which was expanded to include the venous system. The resulting ADAVN (anatomically detailed arterial-venous network) model was a novel 1D closed-loop cardiovascular model that integrated the ultradetailed arterial network of the ADAN with a newly constructed venous network, emphasizing cerebral and coronary territories, and incorporated interactions with cerebrospinal fluid and cardiac mechanics to simulate both normal and pathological conditions. The model comprised 2,185 arterial segments and 189 veins and was validated by comparing the simulation results to hemodynamic data from previously published *in vivo* measurements. Although patient-specific geometries were not used, the model successfully reproduced physiologically realistic pressure and flow waveforms as well as cardiac indices within the expected clinical range.^( 15 )^

Mynard et al **.** developed a model of the systemic and coronary circulation by integrating ventricular pressure, a dynamically modeled aortic valve, and regional coronary flow. The model included approximately 40 arterial segments (systemic and coronary) and was compared with published *in vivo* pressure and flow curves. The model produced realistic pressure and flow curves at multiple sites as well as realistic aortic valve mechanics.^( 16 )^

Olufsen designed a study to improve the physiological accuracy of 1D models of large arteries by introducing a structured tree model at the distal ends of the arterial system, allowing wave propagation effects to persist beyond the truncated computational domain. This would better represent the downstream vasculature than the traditional lumped models. The model included 21 large arterial segments coupled with a structured tree outflow of approximately 17 generations each. This was validated against *in vivo* measurements. The model produced a feasible and physiologically consistent simulation that matched more accurately to *in vivo* data, accounting for wave propagation, impedance, and arterial-tissue coupling at the terminal level.^( 17 )^

Schaaf et al.^( 33 )^ developed a nonlinear 1D model of arterial pulse wave transmission incorporating finite radial wall displacements. The model included 47 arterial segments, and the simulated pressure and flow curves at 14 sites along the arterial tree were compared with published data, showing a good match between the simulated and *in vivo* data. It demonstrated that nonlinear models improved realism over linear and lumped-parameter models.

Stergiopulos et al.^( 34 )^ developed a nonlinear 1D computer model of arterial circulation with 55 arterial segments and used it to investigate the hemodynamic effects of arterial and aortic stenoses. This was validated against published literature and ultrasonographic data, and the produced pressure and flow waveforms were comparable to *in vivo* measurements. The model also accurately reproduced the pulse pressure amplification from the aorta to the femur and the impact of stenoses on the flow, pressure, and pulsatility index.

### Multiscale models (1D±0D/ 3D)

Blanco et al **.** developed a closed-loop computational model of the entire cardiovascular system, incorporating 1D arterial models, the venous system as 0D compartments, and 3D geometry to simulate global and local hemodynamic conditions under physiological and pathological conditions. The model comprised 128 arterial segments and was validated against literature, producing realistic pressure and flow outputs. This model is suitable for simulating complex scenarios and their impact on regional hemodynamics ( *e.g* ., aneurysms). They also stressed the importance of arterial-venous-cardiac-pulmonary coupling.^( 18 )^

Liang et al. developed a multiscale closed-loop model of the cardiovascular system by integrating a 1D arterial tree with a 0D lumped parameter model for the heart, pulmonary, and peripheral circulations. This model was used to investigate the effects of the aortic valve and arterial stenoses on global hemodynamics. It included 55 arterial segments with the heart and veins as 0D compartments. Furthermore, it produced realistic pressure and flow waveforms with adequate systolic amplification, and performed satisfactorily in simulated pathological situations.^( 19 )^

Müller et al **.** presented a global, closed-loop, multiscale model of the human circulation with a detailed 1D description of both arterial and venous systems. The model also included 0D models of the heart, microcirculation, and pulmonary compartments. The model included 85 major arteries and 92 veins in 1D, and a 0D model of the capillaries, heart, and pulmonary compartments. It was validated against MRI-derived flow waveforms in the head and neck veins; literature-based data for arterial system waveforms and pressure flow; and *in vitro* and physiological data for wave speed, pressure-area relations, and venous collapse dynamics. The model produced robust wave simulations that were compatible with the validated methods.^( 20 )^

Mynard et al **.** developed a 1D closed-loop model of the entire adult cardiovascular system that included detailed representations of systemic, pulmonary, coronary, and portal circulations. The circulatory 1D model was coupled with a lumped-parameter heart model that incorporated chamber interactions. It included 396 vessels (arteries and veins), 5,359 nodes, and 188 junctions, and was validated against *in vivo* published data. The model produced realistic pressure and flow curves and accurately captured wave reflections in arterial and venous circulation.^( 21 )^

Reymond et al. built and validated a 1D model of the human systemic arterial tree, including the cerebral and coronary circulation, coupled with a 0D heart compartment. The model included 103 arterial segments and was validated *in vivo* using flow and pressure data from healthy young volunteers. It produced pressure and flow curves that closely matched the *in vivo* measurements, with a mean flow error of approximately 12% and a pressure error below 10% at most locations.^( 22 )^

Safaei et al.^( 32 )^ proposed a comprehensive, open-source computational framework for simulating full-body cardiovascular circulation by integrating 1D, 0D, and 3D models to enable multiscale coupling with organ physiology and biomechanics. The model included 86 arterial segments of the ADAN86 model, along with 230 elements and 457 nodes, and partially incorporated the venous system. It was validated against published physiological data, and successfully reproduced realistic hemodynamic waveforms. Importantly, by relying predominantly on 1D and 0D modeling and reserving 3D components for localized regions, the framework achieved a significant reduction in computational processing time compared with full 3D simulations.

### Comparison of different models

In table 2 , we present a comparison of strictly 1D models regarding their anatomical and physiological fidelity, validation methods, as well as their strengths, and limitations.

**Table 2:** Comparison of different cardiovascular models

Author (year)	Anatomical fidelity	Physiological fidelity	Validation method	Strengths	Limitations
Alastruey et al. (2011)^(^^10^^)^	Medium-high	High	*In vitro* + comparison with elastic model	Pulse wave analysis, viscoelastic effects	Limited network size, no venous system
Blanco et al. (2015)^(^^6^^)^	Very high	High	Anatomical + literature consistency	Detailed anatomical coverage, parametric flexibility	Complex setup, limited *in vivo* validation
Müller et al. (2023)^(^^15^^)^	Very high	Very high	Anatomical + integrated physiology	Closed-loop, detailed arterial-venous network	High computational demand, limited clinical data
Reymond et al. (2009)^(^^22^^)^	High	High	*In vivo* (pressure, velocity)	Validated with real data, good wave behavior	No venous system, smaller network than Müller
Westerhof et al. (2020)^(^^9^^)^	High	High	Developmental physiology literature	Covers full age range (0-20 y), good clinical correlation	No venous network, simplified peripheral modeling

## DISCUSSION

We designed this study to review the literature on computational-assisted 1D models of the entire circulatory system. We identified 19 publications spanning six decades, from 1972 to 2023. Of the 19 studies included, 11 models were nonlinear 1D, six were multiscale and included the venous system, and only two were linear.

Almost two-thirds of the included models were strictly based on the 1D modeling approach. The 1D models are less demanding computationally than the 3D models and are effective for simulating global hemodynamics and producing accurate pressure and flow curves. However, to be feasible, they require simplifications and assumptions, making them less adaptable to complex geometries and limited to simulating pathologies. Complex multiscale models that incorporate 0D and 3D components into 1D models enhance the accuracy and applicability of simulations, particularly for patient-specific analyses and local-level conditions. They provide detailed local hemodynamics and vessel-wall interactions and are highly flexible in adapting to more complex geometries. As expected, this resulted in a higher computational cost, particularly because of the 3D components of the models.^( 23 - 27 )^

Most models included in our review were nonlinear. Nonlinear models perform better than linear models in large elastic arteries, such as the aorta, under highly variable pressure conditions. They are also better suited for simulating pathological conditions. However, linear models operate at lower computational costs and appear to be sufficiently accurate for estimating the flow and pressure in smaller, stiffer vessels with small variations in pressure and operating at lower blood flow rates, especially in lumped models of the circulatory system.^( 28 - 30 )^

Computational costs are of paramount importance when considering 1D versus multiscale models. As stated earlier, 1D models demand less computational power when compared with multiscale models. A full-body arterial tree with over 1,000 arterial segments can be simulated in less than 1 min using a standard laptop computer, whereas a multiscale simulation with 3D coronary segments and a full-body 1D circulatory tree coupled with 0D compartments can take several hours on a high-performance computer (i.e., clusters of very powerful processors working in parallel to process complex operations). Defining the best model depends on the clinical setting, available resources, and spatial resolution required.^( 15 , 23 , 24 , 27 , 31 )^

### Limitations

This study has some limitations. First, the models were heterogeneous, and some articles failed to elucidate which arterial segments were included, why they were included, and how they were modeled. Second, these articles were published over a wide timespan, from the early 1970s to 2023. With regard to computational capacity, scientific research in 1972 relied on mainframe computers, which were large, expensive, and limited in capacity and availability. For instance, a PDP-12 mainframe in 1972 had its memory capacity measured in megabytes with a computational power of approximately 0.01 MIPS (million instructions per second), whereas modern day workstations have their memory capacities measured in petabytes, and an Apple M2 chip has a computational power of approximately 370,000 MIPS. Third, the models were not directly compared, except for ADAN and its reduced version, ADAN86,^( 14 )^which makes multilateral comparisons almost not feasible.

## CONCLUSION

The 1D blood flow models provide a robust and computationally efficient framework for simulating global cardiovascular hemodynamics. Although nonlinear and multiscale models enhance the physiological fidelity and adaptability to complex scenarios, their higher computational demands should be weighed against the available resources and specific clinical or research goals. From a clinical standpoint, 1D and multiscale computational models have shown increasing applicability in cardiovascular medicine. These models have been used to simulate patient-specific hemodynamics in complex cases - such as aortic aneurysms, arterial stenoses, and congenital malformations - aiding in surgical planning and risk assessment. Notably, simplified 1D models have been employed to noninvasively estimate the fractional flow reserve from coronary computed tomography angiography or invasive angiography, reducing the need for pressure wires or hyperemic agents. Multiscale models have also contributed to the device design and evaluation, including stents and grafts, by replicating realistic flow conditions. As computational methods become more accessible and integrated with medical imaging, these models hold promise for personalized diagnoses, virtual surgery simulations, and real-time procedural guidance in interventional cardiology.

## SUPPLEMENTARY MATERIAL

SEARCH STRATEGIES

Table 2S Standard data collection form

**Table:** 

Scoping Review Summary	
Citation	
Study objective	
Model characteristics	
**Feature**	**Description**
Model type	
Wall properties	
Geometry	
Numerical method	
Simulation fluid	
**Model scope**	
• Number of segments:	
• Includes venous system:	
**Validation method**	
Models validated by:	
**Key results from reviewed models**	
**Model / Study**	**Findings**




**Conclusions**	

Table 3S Data extraction and summary of the individual studies

**Table:** 

Alastruey et al. (2011)			
**Citation**			
Alastruey J, Khir AW, Matthys KS, Segers P, Sherwin SJ, Verdonck PR, et al. Pulse wave propagation in a model human arterial network: assessment of 1-D visco-elastic simulations against *in vitro* measurements. J Biomech. 2011;44(12):2250-8.			
**Study objective**			
To assess the accuracy of nonlinear 1D viscoelastic equations of pressure and flow wave propagation in a realistic model of the human arterial system compared with *in vitro* measurements and to evaluate improvements over previous purely elastic models.			
**Model characteristics**			
**Feature**	**Description**		
Model type	1D nonlinear time-domain viscoelastic formulation		
Wall properties	Voigt-type viscoelasticity		
Geometry	1:1 replica of 37 largest conduit arteries in the systemic circulation		
Validation method	*In vitro* measurements with silicone tubes (no data fitting used)		
Numerical scheme	Discontinuous Galerkin with spectral/hp discretization		
Simulation fluid	65% water - 35% glycerol (mimics blood; ρ=1050kg/m ^3^ , μ=2.5mPa·s)		
**Model scope**			
Number of arterial segments: 37 Includes venous system: No			
**Key results**			
**Metric**	**Purely elastic model %**	**Viscoelastic model %**	**Improvement**
Pressure error (EP)	3.0	2.5	p<0.012
Flow rate error (EQ)	15.7	10.8	p<0.002
Harmonic error (flow)	7.0	3.3	p<10 ^-^ ^6^
Harmonic error (pressure)	0.7	0.5	p=0.107 (NS)
**Conclusions**			
Inclusion of the wall viscoelasticity significantly improved the accuracy of the 1D simulations This is especially relevant for reproducing the high-frequency components of pressure and flow waveforms Confirmed the clinical and research utility of 1D models when appropriate physical parameters are known 1D viscoelastic modeling achieved a good balance between accuracy and computational cost			
**Avolio (1980)**			
**Citation**			
Avolio AP. Multi-branched model of the human arterial system. Med Biol Eng Comput. 1980;18(6):709-18.			
**Study objective**			
To develop a physiologically realistic, multibranched 1D computational model of the entire human arterial system—accounting for wave propagation, impedance, and pathological states such as arteriosclerosis and arterial stenosis.			
**Model characteristics**			
**Feature**		**Description**	
Model type		1D transmission-line model (electrical analog principles)	
Wall properties		Viscoelastic (with dynamic Young’s modulus and phase shift)	
Geometry		Multi-branched network of 128 arterial segments	
Numerical method		FORTRAN code using transmission line theory with impedance calculations	
Simulation fluid		Blood modeled using realistic viscosity and density	
**Model scope**			
Number of arterial segments: 128 Includes venous system: No			
**Validation method**			
Comparison of modeled vascular impedance and waveforms with *in vivo* human data: Experimental data from seven patients undergoing cardiac surgery Additional validation with data from previous studies on various arteries			
**Key results**			
**Finding**		**Notes**	
Input impedance matched human data across frequency range		Especially accurate >2 Hz	
Waveforms (pressure & flow) simulated in various segments		Realistic timing, amplitude, and shape	
Pulse wave velocity matched theoretical Moens-Korteweg estimates		approximately 480 cm/s in descending aorta	
Model captured effects of arteriosclerosis		Increased stiffness → ↑ systolic pressure, ↓ time delay	
Simulated arterial stenosis in femoral artery		Detectable only at >65-70% diameter reduction	
Pathological states analyzed: arteriosclerosis, stenosis, wave reflection		Enabled simulations of hypertension patterns and proximal/distal shifts	
**Conclusions**			
The model realistically simulated pressure and flow dynamics in the human arterial tree Provided good agreement with experimental data across frequencies and vascular beds Particularly valuable for pathophysiological simulations, for example, assessing stenosis impact and pulse wave reflections Advantages over analog models—high fidelity to anatomical structures and greater flexibility for parameter changes			
** **Bárdossy et al. (2010)** **			
**Citation**			
Bárdossy G, Halász G. Modeling blood flow in the arterial system. Periodica Polytechnica Mechanical Engineering. 2011;55(1):49-55.			
**Study objective**			
To develop a 1D unsteady viscoelastic model of blood flow in the human arterial network using the method of characteristics (MOC) and a novel Stuart viscoelastic material model capable of simulating pathologies, such as arteriosclerosis.			
**Model characteristics**			
**Feature**		**Description**	
Model type		1D unsteady flow model using the method of characteristics (MOC)	
Wall properties		Stuart viscoelastic model (Kelvin-Voigt + elastic spring)	
Geometry		Network with 45 arterial segments, based on Avolio ^(^ ^11^ ^)^ and Wang et al ^(^ ^8^ ^)^	
Numerical method		Custom-built solver in Transient simulator software (FORTRAN)	
Simulation fluid		Newtonian blood, density=1050kg/m ^3^	
**Model scope**			
Number of arterial segments: 45 Includes venous system: No			
**Validation method**			
Viscoelastic parameters empirically adjusted by comparing them with known pressure waveforms from the literature (Avolio, Wang et al). Additional validation of the Stuart model from previous bench tests with silicone tubes			
**Key results**			
**Finding**		**Notes**	
Increasing systolic pressure along the arterial tree		Diastolic pressure remains relatively stable	
Dicrotic notch observed in aortic pressure wave		Indicates high model fidelity	
Backflow in iliac arteries during diastole captured		Reproduced known physiological behaviors	
Simulated right external iliac stenosis		Diameter reduced from 8.0mm to 2.2mm	
Peak pressure drop with stenosis: 122 → 79mmHg		Diastolic: 77 → 69mmHg	
Flow velocity drop: 0.52 → 0.14 m/s		Backflow disappears with stenosis	
Only 22% flow rate reduction despite 72.8% stenosis		Matches physician observations	
**Conclusions**			
The model accurately reproduced physiological waveforms, including key features such as dicrotic notch and diastolic backflow Capable of simulating local stenosis and arteriosclerosis with segment-specific detail Stuart viscoelastic model adds significant realism over Hookean assumptions Future goals include automated parameter tuning, patient-specific adjustments, and comparison to *in vivo* measurements			
**Blanco et al. (2013)**			
**Citation**			
Blanco PJ, Feijóo RA. A dimensionally-heterogeneous closed-loop model for the cardiovascular system and its applications. Med Eng Phys. 2013;35(5):652-67.			
**Study objective**			
To develop a closed-loop computational model of the entire human cardiovascular system using heterogeneous mathematical descriptions—including 1D arterial models, 0D lumped compartments, and embedded 3D geometries—to simulate both global and local hemodynamics under physiological and pathological conditions ( *e.g.* , aortic regurgitation).			
**Model characteristics**			
**Feature**		**Description**	
Model type		1D-0D-3D heterogeneous closed-loop model	
Wall properties		Nonlinear viscoelastic model (includes damping term)	
Geometry		128 systemic arterial segments (1D); 61 Windkessel terminals	
Numerical method		Finite volume for 1D; Crank-Nicolson and ALE for 0D/3D models	
Simulation fluid		Newtonian, ρ=1.04g/cm ^3^ , μ=0.04 dyn·s/cm ^2^	
**Model scope**			
Number of arterial segments: 128 Includes venous system: Yes (full 0D compartments)			
**Validation method**			
Parameter tuning based on published physiological data. Compared pressure/flow waveforms and valve dynamics with known references			
**Key results**			
**Simulation condition**		**Finding**	
Physiological state		Realistic pressure/flow waveforms, valve dynamics, chamber volumes	
Aortic regurgitation (graded severity)		↓ Aortic pressure, ↑ LV end-diastolic volume, ↑ atrial pressure	
Carotid and cerebral hemodynamics		Inverted diastolic flow and ↓ WSS in aneurysm under severe regurgitation	
WSS/OSI/MRT in aneurysm		↓ WSS by 24%, ↑ OSI by 381%, ↓ MRT by 34% (severe case)	
Backward flow during diastole		Captured in iliac arteries and aneurysms	
System sensitivity		Large hemodynamic shifts emphasize the need for homeostatic controls	
**Conclusions**			
Combined local and global modeling for a realistic simulation of cardiovascular physiology Suitable for simulating complex pathologies and their impact on specific regions (e.g., aneurysms) Highlights the role of arterial-venous-cardiac-pulmonary coupling Model supports patient-specific diagnostics and therapeutic planning Suggests future integration of autonomic control mechanisms ( *e.g* ., baroreflex)			
**Blanco et al. (2014)**			
**Citation**			
Blanco PJ, Watanabe SM, Dari EA, Passos MA, Feijóo RA. Blood flow distribution in an anatomically detailed arterial network model: criteria and algorithms. Biomech Model Mechanobiol. 2014;13(6):1303-30.			
**Study objective**			
To define physiologically accurate criteria for blood flow distribution in a detailed 1D arterial model (ADAN - anatomically detailed arterial network) and develop a numerical calibration algorithm to compute terminal resistances, enabling realistic simulation of regional perfusion across 144 vascular beds (specific organs and distributed territories).			
**Model characteristics**			
**Feature**		**Description**	
Model type		1D arterial network with 144 terminal locations (closed loop)	
Wall properties		Nonlinear viscoelastic wall with elastin, collagen, and smooth muscle	
Geometry		2,142 arteries, including 1,598 named segments + 544 perforator arteries	
Numerical method		Newton-based optimization, least squares FEM with finite differences	
Simulation fluid		Newtonian; μ=4 cP (arteries), 1 cP (perforators); ρ=1.04g/cm ^3^	
**Model scope**			
Number of segments: 2,142 arteries (1,598 named + 544 perforators) Includes venous system: No			
**Validation method**			
Anatomical calibration using vascular territories based on Taylor and other atlases. The waveforms and pressures were validated against the literature. Jacobian-based resistance was optimized using Newton’s method			
**Key results**			
**Component**		**Finding**	
Blood flow to 28 specific organs		64.7% of cardiac output	
Blood flow to 116 vascular territories		35.3% of cardiac output	
Total arteries modeled		2,142 vessels (unprecedented resolution for 1D model)	
Terminal resistances optimized		*Via* Newton method solving 144-equation nonlinear system	
Backflow & waveform fidelity		Captured retrograde flows and realistic pressure/flow profiles	
Coronary arteries		Time-varying terminal pressures from ventricular pressure profile	
Perforator artery resolution		Mapped each vascular territory to source arteries with area-volume flow laws	
**Conclusions**			
Provides a high-resolution arterial network (ADAN) with validated blood flow distribution criteria Model accounts for specific and distributed perfusion using advanced anatomical data Introduced an efficient calibration algorithm for terminal resistance estimation Suitable for regional hemodynamics studies, surgical planning, and spinal cord flow assessments Model and database are publicly available at: http://hemolab.lncc.br/adan-web.			
**Blanco et al. (2015)**			
**Citation**			
Blanco PJ, Watanabe SM, Passos MA, Lemos PA, Feijóo RA. An anatomically detailed arterial network model for one-dimensional computational hemodynamics. IEEE Trans Biomed Eng. 2015;62(2):736-53.			
**Study objective**			
To present the ADAN model—a detailed 1D computational model of the entire arterial system of an average adult man—integrating vascular anatomy, morphometry, wall mechanics, and hemodynamics, validated against both generic physiological data and patient-specific measurements. This model aimed to advance cardiovascular research and clinical planning.			
**Model characteristics**			
**Feature**		**Description**	
Model type		1D model with over 2,000 arterial vessels (ADAN model)	
Wall properties		Nonlinear viscoelastic model (including collagen contribution)	
Geometry		2,142 vessels (1,598 named + 544 perforators), 3D space with anatomical realism	
Numerical method		Finite difference method with Windkessel outflow models	
Simulation fluid		Newtonian incompressible fluid	
**Model scope**			
Number of arterial segments: 2,142 (1,598 named + 544 perforators) Includes venous system: No Blood supply to 28 specific organs and 116 vascular territories			
**Validation method**			
Comparison of pressure and flow waveforms with published *in vivo* data Comparison of cardiovascular indices (CO, ABI, AI, PWV, and PPA) with reference values Patient-specific case: Invasive catheter-based pressure waveforms used for calibration (32-year-old male). Sensitivity analysis: perturbation of arterial stiffness across regional territories			
**Key results**			
**Evaluation component**		**Finding**	
Waveforms in brain, limbs, abdomen		Match *in vivo* recordings across central and peripheral arteries	
Patient-specific calibration		Accurately predicted 6 pressure waveforms along arterial tree	
Impedance analysis		Matches Type B curve, Z _0_=1205 dyn·s/cm ^5^ (close to literature)	
Cardiovascular indices		HR=60 bpm, CO=6.7 L/min, ABI=1.11 (all within normal range)	
Sensitivity analysis		Aortic arch and iliac stiffness greatly impact pressure pulse form	
Comparison with simplified model (55 vessels)		ADAN performs better, especially in the presence of stenoses	
ICA stenosis simulation		ADAN predicts 1.5% ↓ cerebral flow *versus* 12.96% ↓ in simplified model	
Subclavian steal syndrome simulation		Accurately captured retrograde flow in VA and redistribution	
**Conclusions**			
ADAN is the most anatomically realistic 1D model till date, integrating geometry, physiology, and pathology Capable of simulating both generic and patient-specific hemodynamics Demonstrated superior performance *versus* simplified models in pathological conditions ( *e.g.* , stenosis, SSS) Useful in research, education, and potentially in clinical diagnostics and planning Publicly available: http://hemolab.lncc.br/adan-web			
**Blanco et al. (2020)**			
**Citation**			
Blanco PJ, Müller LO, Watanabe SM, Feijóo RA. On the anatomical definition of arterial networks in blood flow simulations: comparison of detailed and simplified models. Biomech Model Mechanobiol. 2020;19(5):1663-78.			
**Study objective**			
To compare the predictive capacity of the anatomically detailed ADAN model (2,142 arteries) with its simplified version (ADAN-86) in both healthy and pathological conditions, particularly for evaluating collateral blood flow during common carotid artery (CCA) occlusion and in the Circle of Willis (CoW) anatomical variations.			
**Model characteristics**			
**Feature**		**Description**	
Model type		1D closed-loop model (ADAN *versus* ADAN-86)	
Wall properties		Nonlinear viscoelastic (elastin, collagen, smooth muscle contributions)	
Geometry		ADAN: 2,142 arteries (4041 1D segments); ADAN-86: 86 vessels (with CoW)	
Numerical method		Finite volume (local time stepping), 10 cardiac cycles simulated	
Simulation fluid		Newtonian; same inflow conditions for both models	
**Model scope**			
Number of segments: ADAN=2,142 arteries; ADAN-86=86 arteries Includes venous system: No			
**Validation method**			
Global cardiovascular indices (CO, PWV, ABI) Impedance and wave intensity analysis Comparison of pressure/flow waveforms in central and peripheral arteries Simulation of left CCA occlusion, with and without anterior communicating artery (ACoA) Benchmarking against values from previous ADAN studies and literature			
**Key results**			
**Comparison aspect**		**ADAN *versus* ADAN-86 findings**	
Healthy scenario		Similar pressure waveforms centrally; large peripheral flow differences	
Carotid occlusion (w/ACoA)		ADAN preserves cerebral flow better; lower pressure drop across occlusion	
Carotid occlusion (wo/ACoA)		ADAN predicts extracranial → intracranial (ECA-to-ICA) steal; more realistic hemodynamics	
Wave intensity analysis		ADAN shows richer forward/backward wave interaction near occlusion	
Flow redistribution		ADAN features extracranial collaterals (thyroid, facial arteries)	
Pressure drop across occlusion		ADAN: 4.0 × 10 ^4^ dyn/cm ^2^; ADAN-86: 10.6 × 10 ^4^ dyn/cm ^2^	
CBF reduction in occlusion (wo/ACoA)		ADAN: -11.7%; ADAN-86: -25.7%	
Steal phenomena		ADAN shows ICA-to-ECA (with ACoA) and ECA-to-ICA (without ACoA)	
**Conclusions**			
Detailed anatomical modeling (ADAN) improved prediction in pathological scenarios, for example, carotid occlusion Simplified models performed adequately in healthy or global hemodynamic studies, but failed to capture collateral pathways The degree of anatomical detail critically determines the accuracy of simulation in disease and variation ADAN enabled investigation of complex hemodynamic effects such as posterior steal, wave reflections, and contralateral compensation			
** **Liang et al. (2009)** **			
**Citation**			
Liang F, Takagi S, Himeno R, Liu H. Multi-scale modeling of the human cardiovascular system with applications to aortic valvular and arterial stenoses. Med Biol Eng Comput. 2009;47(7):743-55.			
**Study objective**			
To develop a multi-scale closed-loop model of the cardiovascular system by integrating a 1D arterial tree with a 0-D lumped parameter model for the heart, pulmonary, and peripheral circulations. This model was used to study the global hemodynamic effects of the aortic valve (AV) and arterial stenoses in various regions of circulation.			
**Model characteristics**			
**Feature**		**Description**	
Model type		Multi-scale model: 1D arterial tree + 0-D heart, veins, pulmonary circulation	
Wall properties		Elastic with nonlinear pressure-area relation (viscoelasticity for heart)	
Geometry		1D: 55 large arteries; 0-D: complete heart and venous blocks	
Numerical method		1D: Lax-Wendroff; 0-D: Runge-Kutta; Interface: ghost-point + Newton-Raphson	
Simulation fluid		Newtonian; ρ=1.06g/cm ^3^, μ=4.43s/cm ^2^	
**Model scope**			
Number of segments: 55 large arteries Includes venous system: Yes (as 0-D compartments)			
**Validation method**			
Comparison of simulated pressure and flow waveforms at multiple anatomical locations using physiological data from literature and clinical standards. Assessment of heart-vascular interactions using an elastance-based cardiac model			
**Key results**			
**Scenario**		**Findings**	
Normal circulation		Realistic pressure/flow waveforms with proper systolic amplification	
AV stenosis (85%)		Prolonged aortic pressure rise, ↑ LV pressure, ↓ SV by approximately 10.5%, ABI unchanged	
Thoracic/abdominal aortic stenosis		Proximal pressure overshoot, ↓ distal pressure, moderate LV impact	
Renal/femoral stenosis		Minimal LV impact, ↓ ankle pressures, reduced ABI<0.92	
Ankle-Brachial Index (ABI)		AV and renal stenoses preserve ABI; others reduce it	
Wave reflections		Significant upstream reflections with aortic stenosis; distal flattening	
**Conclusions**			
Stenosis effects are highly location-dependent; aortic lesions impact the heart whereas distal ones affect ABI Closed-loop integration enables the study of heart-artery coupling and peripheral changes in the same framework The model reproduces clinical findings and offers insight into noninvasive pulse-based diagnostics Useful tool for diagnostic research, studying combined cardiac-vascular pathology, and wave reflection analysis			
** **Müller et al. (2014)** **			
**Citation**			
Müller LO, Toro EF. A global multiscale mathematical model for the human circulation with emphasis on the venous system. Int J Numer Methods Biomed Eng. 2014;30(7):681-725.			
**Study objective**			
To present a global closed-loop multiscale model of the human circulation with a detailed 1D description of both the arterial and venous systems, complemented by 0-D models of the heart, microcirculation, and pulmonary compartments. A special emphasis is placed on modeling venous hemodynamics, especially for the head and neck veins, motivated by links to neurodegenerative diseases.			
**Model characteristics**			
**Feature**		**Description**	
Model type		Multiscale closed-loop model (1D + 0D)	
Wall properties		Variable mechanical properties, nonlinear viscoelastic tube laws	
Geometry		85 major arteries and 92 veins modeled as 1D; full 0D model for capillaries, heart, and pulmonary system	
Numerical method		High-order ADER method; DOT Riemann solver for 1D	
Simulation fluid		Newtonian incompressible fluid, ρ=1.06g/cm ^3^	
**Model scope**			
Number of segments: 85 arteries, 92 veins Includes venous system: Yes (1D venous network, including cerebral veins)			
**Validation method**			
Comparison with MRI-derived flow waveforms in the head and neck veins (patient-specific) Literature-based data for arterial system waveforms and pressure-flow relationships. *In vitro* and physiological data for wave speed, pressure-area relations, and venous collapse dynamics			
**Key results**			
**Component**		**Findings**	
Arterial waveforms		Realistic wave propagation; compliant with literature	
Venous modeling (head/neck)		Captures physiological waveforms; collapse and backflow during upright posture	
Patient-specific simulation		Head/neck venous network calibrated from MRI geometry and flow data	
Numerical scheme		ADER scheme achieved 2 ^nd^-5 ^th^ order accuracy; robust to venous collapse	
Wave speed and stiffness		Veins had wave speeds of 1-3 m/s; stiffness derived from *in vivo* data	
Global hemodynamics		Captures redistribution of flow owing to postural changes	
Riemann problem test		Demonstrated accurate capture of wave reflections and elastic jumps	
**Conclusions**			
This is the first global closed-loop model to feature a detailed 1D representation of the venous system Capable of modeling collapsible veins, transcritical flow, and gravitational effects Patient-specific applications demonstrated using MRI-derived venous geometry and flow Useful for studying neurovascular conditions, e.g., chronic cerebrospinal venous insufficiency The model forms a robust basis for future venous hemodynamic studies and integrated simulations			
** **Müller et al. (2023)** **			
**Citation**			
Müller LO, Watanabe SM, Toro EF, Feijóo RA, Blanco PJ. An anatomically detailed arterial-venous network model. Cerebral and coronary circulation. Front Physiol. 2023;14:1162391.			
**Study objective**			
To develop the ADAVN model (anatomically detailed arterial-venous network)—a novel 1D closed-loop cardiovascular model integrating an ultradetailed arterial system (ADAN) with a newly constructed venous network emphasizing the cerebral and coronary territories, incorporating cerebrospinal fluid (CSF) and cardiac mechanics interactions—to simulate normal and pathological hemodynamics.			
**Model characteristics**			
**Feature**		**Description**	
Model type		Multiscale closed-loop 1D model (arterial + venous + lumped heart and lungs)	
Wall properties		Nonlinear viscoelastic tube laws (different for arteries and veins)	
Geometry		2,185 arterial vessels + 189 veins (including 79 cerebral and 14 coronary)	
Numerical method		Hyperbolized system + ADER finite volume method + local time stepping	
Simulation fluid		Newtonian; ρ=1.04g/cm ^3^; μ=0.04 P (0.01 P in perforators)	
**Model scope**			
Number of segments: 2,185 arteries, 189 veins Includes venous system: Yes (79 cerebral veins, 14 coronary veins, dural sinuses, venous valves, Starling resistors)			
**Validation method**			
Comparison with published *in vivo* measurements of hemodynamic variables Patient-specific geometries and physiological data ( *e.g.* , MRI venous flow) Local sensitivity analysis to assess the venous system impact on cardiac output, ICP, and other parameters Physiological accuracy tested across cardiac and vascular indices			
**Key results**			
**Component**		**Findings**	
Hemodynamic waveforms		Realistic in arteries and veins, reproducing physiological patterns	
Cerebral venous modeling		Includes collapse dynamics and ICP regulation through Starling resistors	
Coronary circulation		Incorporates cardiac muscle compression and myocardial perfusion layers	
Arterio-venous coupling		Flexible multi-capillary connections between arterioles and venules	
Cardiac indices		HR=75 bpm, CO=6.1 L/min, MAP=97.4mmHg (within physiological range)	
ICP regulation		Implemented through dynamic CSF model (Ursino model)	
Venous sensitivity		Venous compliance significantly affects stroke volume and pulse pressure	
Computation		Whole-loop simulation approximately 15 min per cycle with high spatial resolution	
**Conclusions**			
The ADAVN model is the most anatomically and functionally complete 1D arterial-venous model till date Demonstrated the potential for simulating normal and pathological scenarios, with special attention to the brain and heart Incorporated biophysical mechanisms such as collapsible veins, venous valves, ICP, and coronary perfusion Serves as a platform for future investigations in neurovascular diseases, heart-brain interactions, and clinical applications			
** **Mynard et al. (2008)** **			
**Citation**			
Mynard JP, Nithiarasu PA. 1D arterial blood flow model incorporating ventricular pressure, aortic valve and regional coronary flow using the locally conservative Galerkin (LCG) method. Commun Numer Methods Eng. 2008;24(5):367-417.			
**Study objective**			
To develop a comprehensive 1D model of systemic and coronary circulation that integrates ventricular pressure, a dynamically modeled aortic valve, and regional coronary flow using the Locally Conservative Galerkin (LCG) method and to test its behavior under normal, exercise, and pathological conditions.			
**Model characteristics**			
**Feature**		**Description**	
Model type		1D systemic and coronary arterial model with ventriculo-vascular coupling	
Wall properties		Nonlinear elastic law with variable stiffness and taper	
Geometry		Systemic arterial tree with approximately 40 segments; LCA and RCA branch into subendocardial and subepicardial vessels	
Numerical method		Locally Conservative Galerkin (LCG) finite element method	
Simulation fluid		Newtonian; constant density and viscosity assumptions	
**Model scope**			
Number of segments: approximately 40 arterial segments (systemic + coronary) Includes venous system: No			
**Validation method**			
Verification of the waveforms against published *in vivo* pressure and flow patterns Simulation of rest and exercise conditions Comparison of the 1D and 3D velocity profiles in patient-specific carotid bifurcation geometries			
**Key results**			
**Component**		**Findings**	
Systemic waveforms		Realistic pressure and flow waveforms at multiple sites	
Coronary dynamics		Flow suppression in subendocardium during systole; diastolic dominance	
Aortic valve		Opens and closes based on pressure/velocity thresholds; includes RVOT/RVCT	
Afterload sensitivity		Ventricular pressure adjusted by reflected waves and downstream impedance	
Terminal elements		Tapering vessels reproduced realistic input impedance better than Windkessel	
LCG method		Accurate, locally conservative, and efficient with natural branching	
Pathological simulations		Disease features ( *e.g.* stenosis) induced changes matching clinical patterns	
1D *versus* 3D		Good agreement in velocity profiles in carotid bifurcation comparisons	
**Conclusions**			
The model provides a more physiologically accurate framework by integrating realistic aortic valve mechanics, ventriculo-vascular interaction, and regional coronary perfusion LCG method supports complex branching and conserves local flow Offers potential for clinical simulation of disease, exercise hemodynamics, and coronary flow modulation			
** **Mynard et al. (2015)** **			
**Citation**			
Mynard JP, Smolich JJ. One-dimensional haemodynamic modeling and wave dynamics in the entire adult circulation. Ann Biomed Eng. 2015;43(6):1443-60.			
**Study objective**			
To develop a comprehensive 1D closed-loop model of the entire adult cardiovascular system, including detailed representations of systemic, pulmonary, coronary, and portal circulations, coupled with a lumped-parameter heart model incorporating chamber interactions. The model aimed to explore wave propagation, wave intensity, and ventricular-vascular interactions under normal and pathological conditions.			
**Model characteristics**			
**Feature**		**Description**	
Model type		Closed-loop 1D model of full circulation + 0-D heart and microvasculature	
Wall properties		Nonlinear viscoelastic (power law elastic + Voigt viscous)	
Geometry		396 1D segments (arteries + veins) in systemic, pulmonary, coronary, portal	
Numerical method		Finite element method with operator splitting	
Simulation fluid		Newtonian; ρ=1.06g/cm ^3^; μ=0.035	
**Model scope**			
Number of segments: 396 1D vessels, 5359 nodes, 188 junctions Includes venous system: Yes (systemic, pulmonary, portal, coronary)			
**Validation method**			
Comparison with *in vivo* human flow and pressure waveforms Reproduction of waveforms across the aorta, pulmonary arteries, portal system, vena cava, and coronary arteries/veins The hemodynamic variables were validated against published data (CO, SVR, and chamber volumes)			
**Key results**			
**Component**		**Findings**	
Global waveform reproduction		Close match with *in vivo* data for pressure and flow waveforms	
Wave intensity profiles		Simulated wave intensity closely resembled available *in vivo* results	
Aortic/coronary wave behavior		Captured key wave reflection and compression/expansion waves	
Venous wave analysis		Novel insight into wave reflections in vena cava and pulmonary veins	
Cardiac-vascular coupling		Changes in ventricular function influenced waveforms in remote vessels	
Regional differences		Wave dynamics varied across brain, limbs, myocardium, lungs, liver	
Mechanical interactions		Included pericardial pressure, AV plane motion, septal interaction	
Sensitivity analysis		Showed effect of changes in chamber elastance, preload, interaction	
**Conclusions**			
Provides the first anatomically detailed 1D model of full circulation, including systemic, pulmonary, portal, and coronary systems Capable of reproducing global wave dynamics, ventricular-vascular interactions, and regional hemodynamics Offers novel insights into wave reflection, forward/backward wave behavior, and mechanical effects of chamber interactions Useful for investigating cardiovascular disease, wave-based diagnostics, and physiology simulations			
** **Olufsen (1999)** **			
**Citation**			
Olufsen MS. Structured tree outflow condition for blood flow in larger systemic arteries. Am J Physiol. 1999;276(1):H257-68.			
**Study objective**			
To improve the physiological accuracy of the 1D models of large arteries by introducing a structured tree model at the distal ends of the arterial system, allowing wave propagation effects to persist beyond the truncated computational domain. This approach provides a frequency-dependent outflow boundary condition that better represents the downstream vasculature than traditional lumped models.			
**Model characteristics**			
**Feature**		**Description**	
Model type		1D nonlinear model of large arteries + structured tree as outflow condition	
Wall properties		Linearly elastic wall with exponential stiffness-radius relation	
Geometry		21 large arteries + structured binary tree per terminal (∼17 generations)	
Numerical method		Lax-Wendroff scheme; semi-analytical Fourier-based impedance for terminal trees	
Simulation fluid		Newtonian, axisymmetric, laminar flow	
**Model scope**			
Number of segments: 21 large arteries + structured tree outflow (∼17 generations each) Includes venous system: No			
**Validation method**			
Comparison of simulated flow and pressure against measured data from human arteries, Windkessel, and pure resistance boundary models. Evaluated impedance spectra, wave reflections, and pressure-flow phase lag			
**Key results**			
**Component**		**Findings**	
Impedance profiles		Structured tree preserves high-frequency impedance oscillations; better match with human data	
Pressure-flow relation		Maintains physiologic phase lag; avoids artificial reflections	
Comparison with Windkessel		Windkessel fails to model wave reflections and phase shifts	
Reflected waves		Dicrotic notch and wave shape changes captured better with structured tree	
Spatial pressure distribution		Progressive amplification and steeper slopes distally, as *in vivo*	
Outflow impedance		Frequency-dependent convolution relation derived analytically	
**Conclusions**			
The structured tree outflow boundary condition provides a physiologically consistent and computationally feasible alternative to lumped models (e.g., Windkessel) It accounts for wave propagation, impedance spectra, and arterial-tissue coupling at the terminal level Suitable for studies of wave reflection, arterial stiffening, and peripheral resistance Offers flexibility to simulate vasodilation/vasoconstriction by adjusting tree geometry or mechanical parameters Represents a paradigm shift in boundary condition modeling for 1D arterial networks			
** **Reymond et al. (2009)** **			
**Citation**			
Reymond P, Merenda F, Perren F, Rüfenacht D, Stergiopulos N. Validation of a one-dimensional model of the systemic arterial tree. Am J Physiol Heart Circ Physiol. 2009;297(1):H208-22.			
**Study objective**			
To build and validate a comprehensive 1D model of the human systemic arterial tree, including heart-vascular coupling, cerebral and coronary circulation, nonlinear viscoelastic wall properties, and realistic boundary conditions. The model was validated *in vivo* using flow and pressure data obtained from healthy young volunteers.			
**Model characteristics**			
**Feature**		**Description**	
Model type		1D nonlinear arterial model + 0-D heart + Windkessel terminals	
Wall properties		Nonlinear viscoelastic (Holenstein/Bergel)	
Geometry		Detailed full-body arterial tree including coronaries and cerebral circulation	
Numerical method		Implicit finite difference + Newton-Raphson	
Simulation fluid		Newtonian; ρ=1050kg/m ^3^; μ=0.004 Pa·s	
**Model Scope**			
Number of segments: 103 arterial segments Includes venous system: No			
**Validation method**			
Comparison with *in vivo* pressure and flow waveforms in - Ascending, thoracic, and abdominal aorta - Iliac, femoral, carotid, radial, and temporal arteries - Middle cerebral, vertebral, and internal carotid artery Flow *via* PC-MRI and ultrasound Doppler; pressure *via* applanation tonometry			
**Key results**			
**Component**		**Findings**	
Waveform match		Good agreement in pressure and flow waveform shape and amplitude	
Cerebral circulation		Detailed model avoids backflow artifacts, improves physiological pulsatility	
Validation metrics		Mean flow error approximately 12%; pressure error <10% in most locations	
Viscoelasticity		Significant in distal arteries; affects flow and pressure wave shape	
Convective acceleration / WSS		Witzig-Womersley method improves prediction *versus* Poiseuille approximation	
Boundary conditions		3-element Windkessel with impedance matching at terminals	
Coronary modeling		Simplified, coupled to time-varying elastance of LV	
**Conclusions**			
This model is among the most anatomically complete 1D arterial models till date • Includes heart-vascular coupling, nonlinear viscoelasticity, and cerebral/coronary branches Validated against real-world measurements, showing high fidelity in capturing physiological wave dynamics Well-suited for simulating wave propagation, pathologies, and clinical interventions			
**Safaei et al. (2016)**			
**Citation**			
Safaei S, Bradley CP, Suresh V, Mithraratne K, Muller A, Ho H, et al. Roadmap for cardiovascular circulation model. J Physiol. 2016;594(23):6909-28.			
**Study objective**			
To propose a comprehensive, open-source computational framework for simulating full-body cardiovascular circulation—integrating 1D, 0D, and 3D models—and enabling multiscale coupling with organ physiology and biomechanics. This paper outlines the mathematical formulation, software architecture, and validation strategies for large-scale simulations.			
**Model characteristics**			
**Feature**		**Description**	
Model type		Multiscale: 1D arterial model + 0D Windkessel + support for 3D coupling	
Wall properties		Elastic, viscoelastic (Voigt, Maxwell models), with generalized Young’s modulus	
Geometry		ADAN-86: 86 arteries including cerebral, coronary, visceral, limb, and abdominal branches	
Numerical method		Finite element (Galerkin), Crank-Nicolson, CellML-FieldML integration	
Simulation fluid		Newtonian; ρ=1050kg/m ^3^; ν=4 cP (typical)	
**Model scope**			
Number of segments: 86 arterial vessels (ADAN-86 subset), 230 elements, 457 nodes • Includes venous system: Partial support for venous and arteriolar models Coupling to capillaries and tissue beds *via* RCR and CellML-based 0D models			
**Validation method**			
The input flow rate and terminal impedance were modeled using previously published data Terminal impedance was modeled using the RCR Windkessel Validation using pressure/flow waveforms across segments and comparison with published data			
**Key results**			
**Component**		**Findings**	
Pressure/flow waveforms		Physiologic waveforms reproduced across full arterial tree	
Viscoelastic modeling		Maxwell + Voigt + generalized modulus accurately capture wall behavior	
0D-1D coupling		Achieved *via* Riemann invariants; convergence tolerance ε<10 ^-ε^	
Field standards		Implements CellML (ODEs) and FieldML (spatial PDEs) with OpenCMISS	
Vascular territories		Blood flow distribution informed by anatomical perfusion domains	
3D-1D interface		Coupling *via* stress and flow continuity (mass flux, traction)	
Transmission line modeling		Applied in parallel for fast simulations using complex impedance	
Computation time		12 h (1 core); 1.5 h (8 cores); goal=near real-time with future methods	
**Conclusions**			
Presents a blueprint for full-body cardiovascular model incorporating multiscale physics Emphasizes interoperability *via* open standards (CellML, FieldML) and open software (OpenCMISS) Enables simulation of organ-specific perfusion, biomechanical effects, and patient-specific models Sets the stage for future real-time or interactive simulations for clinical or research applications			
** **Schaaf et al. (1972)** **			
**Citation**			
Schaaf BW, Abbrecht PH. Digital computer simulation of human systemic arterial pulse wave transmission: a nonlinear model. J Biomech. 1972;5(4):345-64.			
**Study objective**			
To develop a comprehensive nonlinear 1D model of arterial pulse wave transmission incorporating finite radial wall displacements with improved realism over linear and lumped-parameter models. The model uses a branching arterial tree and captures wave reflections and propagation across systemic circulation.			
**Model characteristics**			
**Feature**		**Description**	
Model type		1D nonlinear arterial model; solved numerically *via* method of characteristics	
Wall properties		Linear elastic (finite radial strain); no viscoelastic terms included	
Geometry		Branching network of approximately 28 major arteries (head, arms, trunk, legs) and 47 arterial segments	
Numerical method		Method of characteristics with finite differences	
Simulation fluid		Newtonian; incompressible; laminar, axisymmetric	
**Model scope**			
Number of segments: 47 arterial segments Includes venous system: No Fourteen output locations mapped to clinically relevant sites			
**Validation method**			
The simulated pressure and flow waveforms at 14 locations were compared with clinical data Fourier analysis was used for the input impedance assessment at the aortic root and femoral artery			
**Key results**			
**Component**		**Findings**	
Waveform fidelity		Good reproduction of clinical pulse wave shapes, inflection points, and amplifications	
Pulse pressure amplification		approximately 70% from aortic root to iliac; within range of clinical data	
Impedance comparison		Better agreement with clinical data than linear models	
Convective acceleration		Shown to be negligible for large vessels	
Wall friction effects		Unsteady friction shown to have minor influence on overall dynamics	
Linear *versus* nonlinear comparison		Linear models produce more oscillatory artifacts; nonlinear model more realistic	
Terminal models		Purely resistive loads used at distal ends	
Computational results		Rapid convergence to physiologic steady oscillations within three cycles	
**Conclusions**			
The nonlinear 1D model captures key pulse dynamics more accurately than linear models Wall distensibility (nonlinear compliance) is crucial in modeling wave propagation, especially in central arteries Convective acceleration and fluid friction contribute little to global wave dynamics Recommended for detailed analysis of arterial wave transmission, especially in elastic vessels such as the aorta Demonstrated that nonlinear modeling provides improved physiological realism			
** **Stergiopulos et al. (1992)** **			
**Citation**			
Stergiopulos N, Young DF, Rowe TR. Computer simulation of arterial flow with applications to arterial and aortic stenoses. J Biomech. 1992;25(12):1477-88.			
**Study objective**			
To develop a fully nonlinear 1D computer model of the systemic arterial circulation and use it to investigate the hemodynamic effects of arterial and aortic stenoses. This model aimed to simulate the pressure and flow waveforms under both normal and pathological conditions and compare the predictions with *in vivo* data.			
**Model characteristics**			
**Feature**		**Description**	
Model type		1D nonlinear model with stenosis module and Windkessel outflows	
Wall properties		Nonlinear compliance using quadratic pressure-area relationship	
Geometry		55 arterial segments (head, arms, trunk, legs)	
Numerical method		Explicit finite difference; upwind differencing; stability Δt<Δx/c	
Simulation fluid		Newtonian, laminar, axisymmetric	
**Model scope**			
Number of segments: 55 arterial segments Includes venous system: No			
**Validation method**			
Comparison with experimental data from the literature (pressure/flow waveforms) Pulsatility index (PI) compared with clinical ultrasound data Segmental systolic pressure index validated against vascular occlusive disease patient data			
**Key results**			
**Component**		**Findings**	
Waveform validation		Good match with clinical waveforms in shape, timing, and amplification	
Pulse pressure amplification		Captures increase from aorta to femoral (approximately 70%), consistent with data	
Effect of stenosis on flow		Critical stenosis severity approximately 85% (normal), approximately 60% (vasodilated state)	
Pulsatility index (PI)		Drops significantly beyond 60% stenosis	
Systolic pressure index		Significant drop in systolic pressure with stenosis >60%; validated in comparison with other studies	
Aortic stenosis simulation		Reproduces delayed rise, reduced amplification, and plateau in periphery	
Stenosis model		Nonlinear pressure drop across lesion modeled using empirical Q-based formula	
Boundary conditions		Three-element Windkessel with realistic impedance matching	
**Conclusions**			
Nonlinear 1D models accurately simulate systemic hemodynamics and pathologic states (arterial and aortic stenosis) Reproduced clinically observed features such as pressure dampening, pulse shape deformation, and wave reflection changes Useful for studying diagnostic markers such as PI and systolic pressure ratios Reinforces the role of computational models in noninvasive diagnostics and physiological interpretation			
** **Wang et al. (2004)** **			
**Citation**			
Wang JJ, Parker KH. Wave propagation in a model of the arterial circulation. J Biomech. 2004;37(4):457-70.			
**Study objective**			
To explore the role of wave reflections and re-reflections in the systemic arterial system using a linearized 1D model of 55 large arteries. This study isolated the effect of arterial geometry on wave dynamics while simplifying the cardiac input and eliminating nonlinearities to better understand pressure and velocity waveforms in health and disease.			
**Model characteristics**			
**Feature**		**Description**	
Model type		1D linearized model of systemic arteries (55 segments)	
Wall properties		Linear elastic (Moens-Korteweg-based wave speed)	
Geometry		Bifurcating tree based previously published data with modified radii	
Numerical method		Method of characteristics with ‘tree of waves’ algorithm	
Simulation fluid		Newtonian; incompressible; U << c assumed (quasi-linear)	
**Model scope**			
Number of segments: 55 arterial segments Includes venous system: No			
**Validation method**			
Waveforms compared with *in vivo* measurements and classic studies Simulated conditions included the heart as absorber *versus* reflector, aortic valve closure, aortic occlusion at four sites, changes in terminal resistance, and coronary flow effects on the reflection coefficients			
**Key results**			
**Component**		**Findings**	
Wave reflection dynamics		Complex re-reflections at bifurcations are main contributors to waveform	
Pulse pressure amplification		Observed distal amplification consistent with clinical data	
Heart reflection effects		Increased pressure in diastole; mimicked aortic regurgitation	
Aortic occlusion modeling		Wave reflections matched experimental canine data previously published data	
Terminal resistance effects		Lower Rp reduced diastolic pressure and reversed flow magnitude	
Coronary circulation		Lowered post-valve reflection coefficient; altered waveform slope	
Transfer function insights		Segment-wise transfer functions contain full info about wave propagation	
Backward wave “trapping”		Reflections attenuated by poorly matched bifurcations on return path	
**Conclusions**			
Wave reflections in a realistic 1D arterial model explained most of the observed pressure and velocity waveform features The “tree of waves” algorithm effectively tracks forward and backward waves with high precision Demonstrated that distal waveform changes and pathological alterations ( *e.g* ., occlusion, regurgitation) can be explained by wave behavior alone Highlights the value of transfer functions and their potential clinical applications, although direct human measurement remains challenging			
** **Westerhof et al. (2020)** **			
**Citation**			
Westerhof BE, van Gemert MJ, van den Wijngaard JP. Pressure and flow relations in the systemic arterial tree throughout development from newborn to adult. Front Pediatr. 2020;8:251.			
**Study objective**			
To develop a distributed hemodynamic model of the human arterial tree that accounts for developmental changes from newborns to adults and to use this model to examine pressure-flow relationships and the applicability of Windkessel models across different ages			
**Model characteristics**			
**Feature**		**Description**	
Model type		1D distributed model with 3-element Windkessel terminals	
Wall properties		Elastic and viscoelastic; wall stiffness and thickness vary with age	
Geometry		121 arterial segments adapted by growth curves of body parts	
Numerical method		Frequency-domain impedance analysis using circuit theory	
Simulation fluid		Newtonian; parameters age-adjusted (flow, heart rate, vessel radius)	
**Model scope**			
Number of segments: 121 arterial segments Includes venous system: No			
**Validation method**			
The simulated pressure and flow waveforms were compared with *in vivo* data from population studies The input impedance and pulse-wave velocity were matched the developmental trends Brachial pressures were validated against clinical centile datasets			
**Key results**			
**Component**		**Findings**	
Pressure amplification		Peripheral pressure in children <5 years ≈ central pressure; amplification increases with age	
Pulse wave velocity		Gradual increase with age; consistent with clinical observations	
Transfer functions		Adult-like shape only from approximately 10 years onward; children <10 years have flatter transfer curves	
Impedance modeling		3- and 4-element Windkessel approximations valid across age range	
Wall shear stress		Preserved constant by scaling radius to tissue perfusion (approximately r ∝ Q^1/3)	
Model calibration		Required adjustments to wall stiffness and peripheral resistance to avoid hypertensive output	
Clinical relevance		Caution advised when using adult transfer functions for children <10 years	
**Conclusions**			
This model provides a comprehensive simulation framework for evaluating arterial hemodynamics from newborns to adults Peripheral-to-central pressure differences are negligible in young children but become significant after approximately 10 years Windkessel models remain applicable if properly scaled by age, height, and body composition This model may aid in interpreting pediatric pressure data, estimating cardiac output, and analyzing the effects of vascular disease or anomalies during development			
